# Resective Surgery for Double Epileptic Foci Overlapping Anterior and Posterior Language Areas: A Case of Epilepsy With Tuberous Sclerosis Complex

**DOI:** 10.3389/fneur.2018.00343

**Published:** 2018-05-17

**Authors:** Tohru Okanishi, Ayataka Fujimoto, Mitsuyo Nishimura, Keiko Niimi, Sotaro Kanai, Hideo Enoki

**Affiliations:** ^1^Department of Child Neurology, Seirei Hamamatsu General Hospital, Hamamatsu, Japan; ^2^Comprehensive Epilepsy Center, Seirei Hamamatsu General Hospital, Hamamatsu, Japan; ^3^Department of Clinical Laboratory, Seirei Hamamatsu General Hospital, Hamamatsu, Japan; ^4^Department of Rehabilitation, Seirei Hamamatsu General Hospital, Hamamatsu, Japan

**Keywords:** epilepsy surgery, tuberous sclerosis complex, language area, awake surgery, language mapping, Broca’s area, Wernicke’s area

## Abstract

Tuberous sclerosis complex is a genetic systematic disorder characterized by hamartomas in multiple organs. Cortical tubers, the hamartomas in the cerebrum, cause multifocal refractory seizures. In certain cases, epileptic foci potentially involve language areas, and hence, extra- and intraoperative cortical mapping can help identify anterior and posterior areas, thus avoiding postsurgical language impairment. We report on a 21-year-old female with tuberous sclerosis complex experiencing refractory partial seizures due to two epileptic foci in the left hemisphere overlapping anterior and posterior language areas. To completely evaluate both language areas, we performed stepwise resections beginning from the anterior to the posterior epileptic focus. Although the patient presented with expressive aphasia following anterior resection, it was possible to conduct language tests during every resection. Postoperatively, she presented with expressive aphasia, comprehension deficits, left-right disorientations, and arithmetic deficits. The language dysfunctions almost disappeared at 5 weeks after the surgery and were completely resolved at 6 months after surgery. At postoperative 9 months, she was free from seizures.

## Introduction

### Patient

#### Clinical Course

A 21-year-old Japanese female, who was right handed and a university student of English literature, presented with multiple hypomelanotic macules since birth. She developed complex partial seizures of speech arrest with twitching in the right corner of her mouth since the age of 7 years. Treatment with carbamazepine and clobazam resolved the seizures. Observations of multiple cortical tubers on magnetic resonance imaging (MRI), facial angiofibroma, and hypomelanotic macules led to a diagnosis of tuberous sclerosis complex (TSC). At age 11 years, her seizures relapsed and were refractory to clonazepam, topiramate, and levetiracetam. At age 20 years, she was referred to our hospital as the seizures began to appear daily and would sometimes evolve to secondarily generalized seizures.

**Figure 1:**
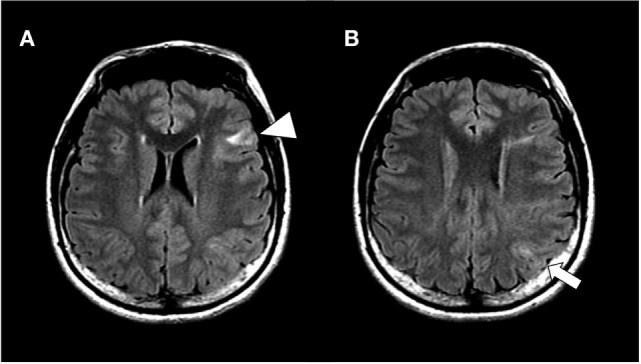
Hippocampal slices of fluid attenuated inversion recovery magnetic resonance imaging at lateral ventricles level **(A)** and next slice above A **(B)**. The patient showed multiple cortical tubers on bilateral hemispheres, including in the inferior frontal gyrus (arrow head) and inferior parietal lobule (arrow).

On first visit to our hospital, her verbal intelligence quotient (IQ), performance IQ, and full IQ were 93, 75, and 83, respectively, on assessment with the Wechsler Adult Intelligence Scale-III. MRI revealed multiple cortical tubers on bilateral hemispheres, including tubers in the inferior frontal gyrus and inferior parietal lobule (Figure 1). 18F-fluorodeoxyglucose-positron emission tomography (PET) showed low-uptake areas corresponding to the cortical tubers, whereas C-11-methionine PET did not show any high or low-uptake areas. During 48 h of monitoring, scalp video-electroencephalography (vEEG) recorded 19 seizures, which were complex partial seizures of speech arrest with twitching on the right corner of mouth and drooling, which was sometimes followed by head turning to the right and secondarily generalized tonic–clonic seizures.

**Figure 2:**
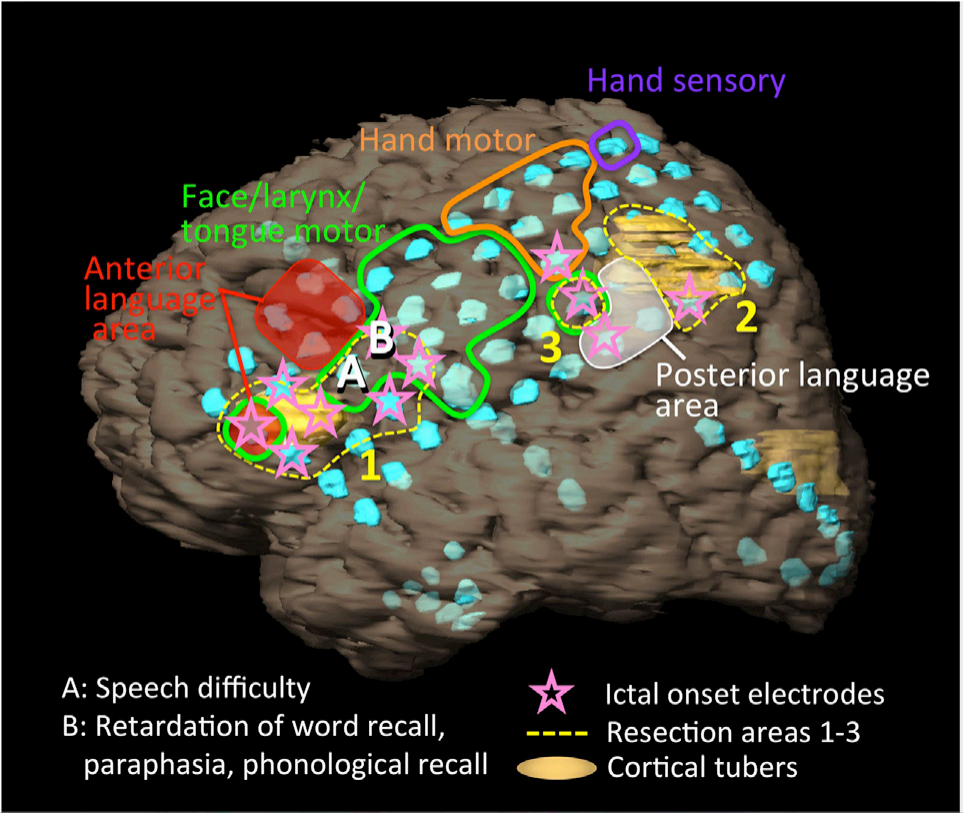
Schema of the patient’s left hemisphere, cortical tubers (yellow regions), intracranial electrodes (light blue disks), results of extraoperative functional mapping of anterior language area (red shaded), posterior language area (white shaded), motor area of face/larynx/tongue (green loop), hand (orange loop), and hand sensory area (purple loop), ictal onset electrodes (pink stars), and resection areas (yellow dot loops: 1–3). We implanted 92 electrodes [2 grid electrodes (8 × 5, 4 × 5), 3 strip electrodes (10, 8, 8), and 1 depth electrode (6)]. One cortical tuber was present below and over the anterior language area. Another cortical tuber distributed posterior to the posterior language area. Ictal onset electrodes partially overlapped with the anterior and posterior language areas. The resections were performed from the resection areas of 1, 2, to 3. During the resection surgery, the patient presented with speech difficulty after the resection of site A, and retardation in responsive naming, paraphasia, and phonological recall after the resection of site B.

We conducted an intracranial EEG analysis (EEG-1200, Neurofax, Nihon Kohden), using 92 electrodes [2 grid electrodes (8 × 5, 4 × 5), 3 strip electrodes (10, 8, 8), and 1 depth electrode (6)] (Figure 2). The grid electrodes covered two cortical tubers in the inferior frontal lobe and inferior parietal lobule and the anatomical anterior language area (Broca’s area: pars triangularis and pars opercularis) and a part of posterior language area (Wernicke’s areas: supra temporal gyrus, supramargnial gyrus, and angular gyrus) in her left hemisphere.

We performed extraoperative (presurgical) language mapping with a stimulation of 4 mA (motor/sensory) or 10 mA (language) at 50 Hz for 1–5 s. We tested language functions using token test, picture naming, and responsive naming. We identified anterior language area at five electrodes on the middle and inferior frontal gyrus that excluded pars triangularis and pars opercularis, and posterior language area at three electrodes in the inferior parietal lobule including the supramarginal gyrus and angular gyrus. Although she represented retardations or disruptions at picture naming and responsive naming and mistakes at token test in both language areas, more severe disturbances and paraphasia occurred during the stimulations to posterior language area (Figure 2, red and white shaded areas).

Intracranial vEEG monitoring was performed for 120 h. Thirty-five partial seizures with/without secondary generalization that started with right mouth twitching and over 50 of electrographic seizures were captured. The ictal fast activities started separately and simultaneously in the frontal and parietal regions (Figure 2, pink stars). The frontal fast activities were distributed over pars triangularis and pars opercularis, which partially overlapped anterior language area and a cortical tuber. The posterior fast activities distributed to the posterior term of the middle frontal gyrus, middle anterior and posterior central gyri, inferior parietal lobule, which partially overlapped the posterior language area and a cortical tuber.

We then performed resection surgery. During the craniotomy, we anesthetized the patient using sevoflurane. She was allowed to recover from the anesthesia until she was conscious enough to undergo a language examination.

Initially, the anterior region from the frontal cortical tuber was resected (Figure 2, resection area 1), and we evaluated language function using electrical cortical stimulation (ECS; biphasic stimulation on each pair of adjacent contacts, 8–12 mA, pulse width 0.1 msec, 50 Hz, 1–5 s) during the cortical resections. We tested language functions using free conversation, picture naming, and responsive naming. She presented with speech difficulty following resection of site A (Figure 2), and retardation of responsive naming, paraphasia, and response in English to a picture naming task after resection of site B. ECS revealed that these sites did not represent language functions. We then resected the posterior regions (Figure 2, resection areas 2 and 3) that extended to the posterior language area. Here, responsive naming tasks were mainly used instead of picture naming. Variable comprehension deficits additionally appeared on resection of area 2. We avoided resection of the posterior language area, which was confirmed *via* extraoperative mapping.

After the surgery, she presented expressive aphasia, including phonemic and verbal paraphasia, disturbed recalling and repetition, neology, agrammatism, prolonged speech, and dysgraphia. She also showed left-right disorientations and arithmetic deficits. She did not show any palsy in her face or extremities. The Standard language Test of Aphasia (SLTA) (1) revealed a 72% decline in total score, with declines especially in following oral commands (20%), repeating verbal sentences (40%), writing Kanji (Chinese characters) words (40%), and dictating sentences (20%).

During 4 weeks after the surgery, she showed gradual improvement in language function and, eventually, her aphasia almost disappeared. Her left-right disorientations and arithmetic deficits had also disappeared until 4 weeks after surgery. The SLTA scores markedly improved by 98%, and she was discharged at 5 weeks after surgery. Furthermore, the SLTA scores recovered completely (100%) at 6 months after the surgery and postoperatively at the 9-month follow-up, she was free from seizures.

## Background

The cortical tubers in tuberous sclerosis complex are capable of inducing epileptogenesis and epileptic activities originate from neural tissues within or surrounding the cortical tubers (2, 3). A retrospective study in the United States conducted in 2010 showed that 25% of TSC patients with refractory partial epilepsy underwent resection surgery (4).

Cortical resection of epileptic foci is a radical surgery for a patient with medically refractory partial seizures (5). However, in cases where epileptic foci overlap eloquent cortices of language function or motor/sensory functions, extraoperative or intraoperative functional mapping of cortices is necessary to avoid neurological sequelae.

Anterior (Broca’s) and posterior (Wernicke’s) language areas are classical representatives of language areas. Anterior language area was considered to be associated with expressive language function and distribute across Brodmann’s areas 44 and 45, while posterior language area was considered to be associated with comprehension and understanding language, and correspond to Brodmann’s areas 22 and 39 (6–8). Recent studies on the associations between cerebral injuries and language functions revealed that the language areas generally extend to middle frontal gyrus, sensorimotor area, posterior temporoparietal area, anterior and posterior temporal areas, and the sites other than cortices, including the basal ganglia, arcuate fasciculus, and superior longitudinal fasciculus and that these areas are associated with impairments in speech comprehension and production (6, 8, 9).

In cases where the epileptic foci potentially include language areas, extraoperative and intraoperative cortical mappings can be used to identify these areas. In extraoperative mapping, intracranial grid electrodes, which are used for intracranial vEEG, are applied for conducting ECS on the possible language areas. Furthermore, in cases of awake surgery, an intraoperative language test is performed concomitantly with intraoperative ECS and cortical resections. It is important to restrict the number of intraoperative language tests owing to limitations in surgical time and to prevent stress for the patient.

## Discussion

We successfully performed multiple resections of epileptic foci, which extended separately to anterior and posterior language areas in a 21-year-old patient with TSC, without permanent language sequelae. Multiple steps of preoperative and intraoperative examinations and stepwise resections from anterior to posterior regions led to the successful results.

Epileptic activity alters conventional language areas in patients with epilepsy (10, 11). In the patients with lesions involving the left inferior or middle frontal gyri, atypical language areas are noted (12). In TSC patients, cortical tubers and epileptic activities in language related cortices also alter the distributions of usual language areas to bilateral hemispheres (13). Although these previous studies indicated the possibility of bilateral language areas, the Wada test and extraoperative mapping revealed motor and language areas approximate to the usual distributions observed in our patient.

In our patient, the epileptic foci were adjacent to or overlapped with anterior and posterior language areas. The language deficits were more severe at the stimulation to the posterior area than it to anterior area. Initiating resection from the posterior language area would severely disrupt her semantic understanding, thus making it difficult to evaluate the functions in the anterior resection area. Therefore, we initiated the resections from the anterior to the posterior areas. During the anterior resection, she presented with expressive aphasia at a cortical site, where we did not identify language areas by extra- and intraoperative electrical stimulations. In a large study analyzing the correlations between preoperative language tests and the language outcomes in the patients with glioma, the resections to the frontal, parietal, or temporal cortices without language functions at extraoperative mapping sometimes cause language deficit after the surgery. Most of the deficits resolve within 6 months (14). Consistent with this study, her language functions also completely recovered as observed during the 6-month follow-up. Damage to motor/sensory areas and arcuate fasciculus between anterior and posterior language areas possibly result in permanent language deficits with respect to speech comprehension or production (6, 9). This patient fortunately did not have epileptic foci in these regions.

This study had some limitations. The patient did not show additional language deficit during the posterior resection. She may have been able to perform the language tasks during the surgery if we had initiated stepwise resections from the posterior region. Further, intraoperative ECS did not identify the language function. The utility of the stimulation cannot be discussed in this case.

## Concluding Remarks

We experienced a patient with TSC who developed two epileptic foci adjacent to or overlapping anterior and posterior language areas. Permanent language deficits were avoided by using functional mapping, stepwise surgery from anterior to posterior regions, and using intraoperative language tests to evaluate language function. In the future, studies on surgical cases with complicated distributions of epileptic foci in the language dominant hemisphere will be required to confirm the validity of stepwise resections.

## Ethics Statement

Written informed consent to publish the report was obtained from the patient.

## Author Contributions

TO contributed to conceptualizing, drafting and revising the study, analyzing, acquiring, and interpreting the data. AF contributed to conceptualizing and revising the study, acquiring, and interpreting the data. MN and SK contributed to the interpretation of EEG data. KN contributed interpreting language test data. HE contributed to conceptualizing and revising the work.

## Conflict of Interest Statement

The authors declare that the research was conducted in the absence of any commercial or financial relationships that could be construed as a potential conflict of interest.
